# Burnout and Coping Strategies in Integrative Psychotherapists: Findings from Qualitative Interviews

**DOI:** 10.3390/healthcare12181820

**Published:** 2024-09-11

**Authors:** Panagiota Tragantzopoulou, Vaitsa Giannouli, Anna Filippou, Margarita Demirtzidou

**Affiliations:** 1 School of Social Sciences, University of Westminster, London W1B 2HW, UK; 2 School of Psychology, Aristotle University of Thessaloniki, 54124 Thessaloniki, Greece; giannouliv@hotmail.com; 3 Mediterranean College, University of Derby, 54625 Thessaloniki, Greece; a.filippou@mc-class.gr (A.F.); m.demirtzdou@mc-class.gr (M.D.)

**Keywords:** burnout, psychotherapists, integrative therapy, coping, qualitative interviews

## Abstract

Burnout among psychotherapists is a pervasive challenge affecting both professional well-being and client care. This study aims to explore the experience of burnout among integrative psychotherapists and examine the strategies they employ to cope with this phenomenon. Interviews were conducted with 17 integrative psychotherapists, and the data were analyzed using Braun and Clarke’s six-step thematic analysis. Through this analysis, two themes were identified: (1) work-related pressures and burnout manifestations and (2) strategies for maintaining optimal functioning. Fatigue, headaches, challenges in decision-making or session planning, numbness in the form of paralysis, and disconnection from clients emerged as primary symptoms, impacting therapeutic efficacy. Participants’ intense sense of responsibility toward clients and their self-worth validation through client progress intensified burnout risks, particularly among novices. Personal therapy and clinical supervision emerged as pivotal in mitigating burnout, offering support, and enhancing therapist resilience. Additionally, peer support and organizational interventions were deemed crucial during crises, emphasizing the need for structured support systems within professional bodies. These findings contribute to a deeper understanding of burnout in psychotherapy and highlight the need for targeted interventions to enhance professionals’ resilience and sustain effective client outcomes.

## 1. Introduction

The psychotherapeutic profession imposes unique emotional challenges on therapists and is characterized by high psychological distress due to the intensive demands of providing empathy and support to clients [1]. Consequently, numerous studies have indicated that a significant proportion of psychotherapists experience burnout at some point in their careers [2,3,4]. Burnout is conceptualized as a form of workplace stress that leads to feelings of exhaustion, mental distance through the development of negative attitudes toward one’s work, and a reduction in professional efficacy [5]. More recently, burnout has been reconceptualized as a syndrome encompassing four primary dimensions: exhaustion, mental detachment, and difficulties in both cognitive and emotional functioning [6]. This new understanding suggests that the previous burnout conceptualization may be flawed, and a more comprehensive framework of burnout is needed. Several factors have been found to be associated with burnout in the psychotherapeutic profession. A strong and consistent theme in the literature indicates that organizational factors—such as heavy workloads, insufficient resources, lack of support, and limited length of therapy career—can negatively affect the well-being of practitioners [3,5]. Particularly among novice psychotherapists, having less experience but excessively high and unrealistic expectations regarding the extent of the support and the level of engagement required in their roles has been linked to burnout [7]. These unmet expectations can result in feelings of low personal achievement, which can significantly trigger burnout [7].

In addition to the common risk factors inherent to their professional context, psychotherapists faced unprecedented challenges during the COVID-19 pandemic, which increased reported burnout rates [4,8]. The public health crisis, coupled with prolonged lockdowns, heightened the population’s distress, leading more individuals to seek professional support [9]. As psychotherapists managed the newly experienced distress of the lockdown and health crisis, they also had to navigate significant shifts in their profession. Transitioning to online sessions proved challenging for some, particularly in terms of accepting the new format while simultaneously handling their own personal and familial challenges. Aafjes-van Doorn et al. [10] found that therapists experienced greater professional self-doubt during the pandemic compared to pre-pandemic levels. This increase in self-doubt was associated with lower acceptance of the online therapy platform, heightened secondary traumatic stress, weaker therapeutic alliances with patients, and limited clinical experience [10]. Nonetheless, only a small proportion of participants were involved in the follow-up measurements, and this study relied solely on quantitative data without exploring the therapists’ experiences in depth.

While numerous studies have investigated burnout among psychotherapists, there is a significant gap in research focused on how these professionals cope with burnout [11]. Although the negative impact of the psychotherapeutic profession on mental health is well established, there is insufficient understanding of the occupational factors that may escalate psychotherapists’ vulnerability to burnout, as well as the strategies that can be used to manage burnout. Research on intrapersonal factors affecting psychotherapists’ professional functioning has highlighted the importance of personality traits and self-care behaviors, such as self-compassion and mindfulness, in maintaining a high quality of life in this profession [4,12,13]. This suggests that prioritizing self-care is essential yet remains significantly underexplored, both in empirical research and in the context of training programs across therapeutic approaches such as the integrative approach [14]. Occupational factors, such as personal therapy, supervision, and support from team co-workers, have further been suggested as buffers against burnout, providing therapists with a safe space for guidance and assistance [15,16]. Therapists often experience anxiety and self-doubt [17,18], which can exacerbate burnout. Therefore, having access to professional support can help therapists manage their stress, ultimately enhancing their professional effectiveness and personal quality of life.

Therapists, particularly integrative therapists, are a group that faces unique stressors but is often overlooked in research. Investigating the burnout experiences and practices for coping and optimizing functioning among therapists and other professionals is crucial, as these factors can influence not only the professionals themselves in terms of self-doubt, traumatic stress, and overall functionality but also the quality of care they provide to their clients. Understanding and supporting therapists’ mental health is crucial for preserving both their personal stability and the effectiveness of the care they deliver. Consequently, the aim of this study is to explore the experiences of burnout and the strategies for coping with burnout among integrative psychotherapists. The research questions this study seeks to answer are as follows: (a) How do integrative psychotherapists perceive and experience burnout in their professional practice? and (b) What strategies do integrative psychotherapists use to cope with burnout? By examining both the challenges they face and the coping mechanisms they employ, this research aims to provide a deeper understanding of the factors contributing to their professional effectiveness.

## 2. Materials and Methods

### 2.1. Design

A qualitative design was adopted for this study to explore the experiences of burnout and the strategies psychotherapists employ to cope with it. Qualitative research is essential for an in-depth investigation of individuals’ experiences and perspectives, providing rich and detailed insights into their personal and professional lives [19].

### 2.2. Sampling and Recruitment

Purposive sampling was utilized to recruit participants with specific characteristics relevant to the study. The criteria for participation included (a) being trained in and currently practicing integrative therapy and (b) having experienced burnout symptoms in the past. An advertisement for the study was posted on social media platforms, leading potential participants to contact the research team. Interested participants received detailed information about the study and were provided with consent forms. They were asked to sign informed consent, ensuring they were fully informed about the study’s purpose, the use of their data, and the process for withdrawing consent. The final sample consisted of 17 integrative psychotherapists, comprising 14 women and three men, aged between 25 and 56 years, with an average professional experience of 8.21 years. The majority of participants were Greek, with one Dutch and one Serbian participant (see Table 1).

### 2.3. Data Collection

Once participants responded to the invitation, one-on-one semi-structured interviews were conducted between September 2023 and December 2023. These interviews were scheduled at times convenient for each participant and were conducted online via a secure video conferencing platform to accommodate geographic diversity and ensure flexibility. Each interview lasted approximately one hour. The semi-structured format allowed the researchers to capture not only the reactions and behaviors of the participants but also their thoughts, feelings, interpretations, and understandings regarding their experiences of burnout and coping strategies. An interview guide was developed specifically to address the research questions and gain a comprehensive understanding of burnout and coping strategies among integrative psychotherapists. This guide included questions designed to explore the experience of burnout, such as “How do you experience burnout?” and its impact on professional practice, with questions like “How has burnout affected your work with clients?”, “What changes have you noticed in your approach to therapy as a result of experiencing burnout?”, and “How has burnout impacted your motivation and job satisfaction?”. Additionally, the guide sought to uncover strategies for managing burnout through questions such as “Can you describe the personal strategies you use to cope with burnout in your professional practice?” and “What practices do you find most effective?”. To facilitate deeper exploration, prompts were used to encourage elaboration on initial responses, including inquiries like “Can you provide an example?” and “Can you describe a specific strategy that you find helpful?”. These prompts aimed to draw out detailed examples and further insights into the participants’ experiences and coping mechanisms. Open-ended questions allowed integrative psychotherapists to thoroughly describe their experiences.

Prior to each interview, participants were reminded of the study’s purpose, their rights as participants, including their right to withdraw at any point, and the measures in place to ensure their confidentiality. Participants were asked to select a pseudonym for anonymity, and the interviews were audio-recorded with their consent. No participants opted to withdraw from the study, and all identifiable information was omitted to ensure confidentiality. At the conclusion of each interview, a debriefing session was conducted, during which participants were given the opportunity to ask questions, express any concerns, or share additional thoughts about the study. Participants were reassured of their confidentiality and reminded of how to contact the research team should they have further inquiries or wish to receive a summary of the study’s findings.

### 2.4. Data Analysis

The data were analyzed using thematic analysis, following a structured and systematic approach. The process began with an in-depth engagement with the interview recordings, including multiple listenings to ensure a thorough understanding of the data. Each recording was transcribed verbatim to capture the nuances of the psychotherapists’ experiences accurately. In the initial phase of coding, preliminary patterns and ideas were identified. Initial codes were developed inductively by identifying significant segments of text that were pertinent to the research questions. These initial codes, both semantic and latent, were generated based on recurring concepts and patterns observed across the interviews. Subsequently, these codes were systematically collated and reviewed. Codes that reflected similar concepts were grouped together into broader categories, which were then organized into themes. Each theme was defined and refined through an iterative process, where the consistency of themes with coded extracts and the entire dataset was checked. Themes were then refined and named while quotes representing the experiences of integrative psychotherapists were included.

### 2.5. Trustworthiness and Rigor

To ensure the trustworthiness and rigor of the findings, meticulous records were diligently kept throughout the analysis phase, encompassing interview transcripts, field notes, and coding procedures. Three of the researchers independently coded the data, followed by collaborative discussions to align on identified themes and interpretations, thereby enhancing credibility. Confirmability was reinforced through the inclusion of pertinent quotes to exemplify emerging themes. Throughout the study, continuous reflexivity was employed, with researchers documenting personal reflections, particularly as three of the researchers were integrative psychotherapists themselves. By transparently acknowledging biases and consciously mitigating their influence during data collection and analysis, we aimed to strengthen the objectivity and reliability of our findings. Additionally, peer debriefing sessions played a pivotal role in stimulating critical discourse on interpretations and decisions, thereby bolstering the methodological rigor of the research.

### 2.6. Ethical Considerations

The study received approval from the researchers’ University Ethics Committee. Participation was voluntary, and all participants provided written consent. Pseudonyms were assigned, and all identifiable data were removed to ensure anonymity and confidentiality. Participants were informed of their right to withdraw from the study at any time, and a debriefing session was conducted for each participant.

## 3. Results

### 3.1. Work-Related Pressures and Burnout Manifestations

This theme highlights the perceived pressures of meeting clients’ needs, proving self-worth, and managing increased workloads, which were described by therapists as escalating burnout symptoms such as fatigue and discomfort during sessions. The theme is divided into two sub-themes: “Professionals Stressors” and “Burnout Symptoms”.

#### 3.1.1. Professional Stressors

A strong sense of duty and responsibility for their clients’ treatment was described by the majority of participants as a key stressor. They recognized that their clients often have significant needs, which intensified their obligation to “be there” for them and maintain a “supportive role” in their lives. The pandemic period, in particular, emerged as a critical juncture for many therapists, as they were inundated with requests from new clients seeking professional support. This period was viewed as being marked by an overwhelming desire for connection, as people turned to therapy to fulfill their need for human interaction during a time of isolation and uncertainty. One participant remarked:

“People wanted to talk… they had an incredible need to feel connected, and it was a motivation to start therapy”.

This surge in demand was described as heightening therapists’ sense of responsibility, which, while validating their role, also was thought to contribute significantly to burnout symptoms. Participants frequently mentioned that clients facing “extreme challenges or life dead-ends” exacerbated their sense of obligation. The weight of these situations compelled therapists to explore “various ways and effective interventions” to provide relief or offer alternative solutions that might foster a more positive outlook in their clients. However, this deepened sense of duty often pushed participants beyond the traditional boundaries of the therapeutic relationship, leading to a level of engagement that sometimes felt overwhelming. The pressure to continuously meet these intensified client needs, especially during the pandemic, not only increased their workload but also blurred the lines of professional boundaries, contributing to a growing sense of fatigue and discomfort during sessions.

“This heightened sense of responsibility comes from seeing people struggling with their lives and wanting to be there to support and guide them through these barriers. This can sometimes lead to becoming overly engaged…even overstepping professional boundaries”.

Beyond the sense of duty and responsibility, many participants also reported that their self-worth was closely tied to their professional effectiveness, particularly among novice therapists with less than five years of experience. For these individuals, the ability to provide effective treatment was seen not only as a professional obligation but also as a measure of their competence and identity as “good therapists”. One participant expressed this sentiment, stating, “If I can’t help my clients, I question whether I’m good enough to be in this profession”.

This strong association between responsibility and self-worth often intensified the pressure they placed on themselves. The desire to demonstrate their capability and prove that they had the necessary skills to succeed in the field frequently drove them to exceed normal expectations, taking on additional clients or extending their availability beyond typical working hours. This relentless pursuit of excellence, while rooted in a genuine commitment to their clients’ needs, often came at a significant personal cost. Many found themselves neglecting their own self-care and boundaries in an effort to meet the high standards they set for themselves:

“It was important for me not only to help my clients but also to feel capable. I would stay up late at night reading or planning the next sessions. Sometimes, I would even spend the whole night thinking without being able to sleep. The next day, I would continue with my maternal responsibilities and my other sessions…this affected my sleep”.

#### 3.1.2. Burnout Symptoms

As work demands escalated, particularly during the COVID-19 period, most participants reported experiencing a range of burnout symptoms. The relentless pressure of their workload manifested physically for many, with participants frequently describing persistent tiredness, headaches, weight loss, and sleep disturbances. One participant shared, “By the end of the day, I felt utterly drained. It was like my body couldn’t keep up anymore”. This pervasive exhaustion often left them feeling overwhelmed, struggling to keep pace with their responsibilities. Another participant echoed this sentiment, stating: 

“It was really difficult to handle everything at the end of the day”.

The cognitive impact of burnout was also a prominent concern. Participants reported a significant decline in their ability to concentrate, make decisions, and engage creatively in their therapeutic work. One therapist reflected on this decline, saying, “I found myself stuck, unable to think of new approaches or interventions. My mind just went blank when I needed it most”. This mental fog and stifled creativity not only hindered their effectiveness in sessions but also contributed to a growing sense of inadequacy and frustration.

Emotionally, the strain of burnout led to a noticeable shift in how participants related to their clients. Several therapists described a disturbing sense of detachment, where they felt increasingly disconnected from their clients’ experiences and emotions. This detachment sometimes evolved into a deeper, more troubling numbness in the form of paralysis, which was experienced as losing control of the body. One participant described this sensation vividly:

“I would feel numb, like freezing inside the session…feeling paralyzed. This strong feeling of disappointment and discomfort would make me want to say, ‘Okay, I think we are done for today’”.

The emotional numbing and detachment were further exacerbated by the development of negative attitudes toward clients. Some participants reported feeling frustrated or even resentful toward their clients, particularly when they felt unable to help them effectively. This negative shift in attitude sometimes led to a desire to terminate sessions prematurely, as one therapist admitted,

“There were moments when I just wanted to end the session early. I felt like I had nothing left to give”.

### 3.2. Strategies for Maintaining Optimal Functioning

This theme captures the various strategies participants discussed to manage burnout. These strategies span from seeking professional assistance to making lifestyle adjustments. Three distinct sub-themes were identified: “personal therapy and clinical supervision”, “inner dialogue and personal development”, and “wellness and relaxation practices”.

#### 3.2.1. Personal Therapy and Clinical Supervision

Personal therapy and clinical supervision were discussed by therapists as essential components in managing burnout. The majority of participants expressed the view that, in cases of burnout, “personal therapy and clinical supervision sessions should be increased while client sessions should be decreased”. This approach was thought to allow therapists to establish boundaries, protect both themselves and their clients, and explore their own fears and symptoms. Most participants regarded personal therapy and clinical supervision as crucial support mechanisms, with the former providing expertise in understanding their personal experiences and the latter “offering guidance on managing burnout and client sessions effectively without compromising client safety”. Moreover, some participants highlighted that engaging in both personal therapy and clinical supervision facilitated a deeper understanding of their own humanity, acknowledging that even psychologists can face challenging periods and uncertainty. This realization reinforced the importance of seeking support:

“We are humans after all…Yes, we are psychotherapists trying to help people, but we are also humans who might go through difficult times. My personal therapy and clinical supervision helped me see this exact thing. I am human just like everyone else, and I may struggle and need support”.

#### 3.2.2. Inner Dialogue and Personal Development

A significant number of participants emphasized the importance of maintaining a continuous inner dialogue and engaging in self-reflection as part of their strategy to cope with burnout. This inner dialogue involved closely listening to their own bodies and minds, paying attention to physical and mental signals, and recognizing early indicators of burnout, such as changes in weight, persistent fatigue, or unexplained aches. These physical and mental cues were described as critical prompts for the need to “take a step back” and reassess their current workload and stress levels. As one participant noted:

“You need to listen to your own body and mind… the thoughts it generates. It’s the only way to step back, recognize the need for setting boundaries, and safeguard yourself by dialing down the pace”.

Beyond simply identifying burnout, participants described this self-awareness as a proactive strategy to protect their mental health. They found that by tuning into their internal state, they could make conscious decisions to adjust their workload, seek additional support, and prioritize self-care practices. This process of inner dialogue was seen not just as a response to adversity but as a key element in preventing burnout recurrence. Participants also highlighted personal development as an essential protective measure against burnout. This development involved not only addressing current challenges but also enhancing their overall professional and personal development. Many cited further education, participation in seminars, and engaging in extensive reading as valuable avenues for broadening their knowledge and skills. Interestingly, one participant shared that they had explored botany as a way to learn more about herbs and their potential use in alleviating burnout symptoms. This exploration of botany was not merely a hobby but a deliberate effort to find natural remedies that could support them: 

“I started learning about different herbs and trying them out to see if they could help me manage it better”.

Moreover, participants stressed the importance of interaction with fellow therapists, particularly those facing similar challenges, as an opportunity for “shared learning”. Engaging with peers in professional development settings or informal discussions was viewed as a crucial strategy for both personal development and emotional support. They also suggested the establishment of support groups for therapists, organized by professional bodies, as a formal mechanism to facilitate knowledge sharing and offer emotional support. One participant reflected on the challenges during the pandemic, stating:

“With the pandemic, this lack of support and knowledge to handle the situation was unbearable. We all seek connection, and having professional support groups for therapists could provide both education and support”.

#### 3.2.3. Wellness and Relaxation Practices

Lifestyle changes were also regarded as a catalyst for managing burnout. Many participants discussed adopting healthier eating habits and incorporating regular exercise into their routines as a means to decompress and establish a healthy lifestyle. Creating a functional routine that included hobbies, physical activity, and nutritious food was described as essential for maintaining “good mental health, stability, and balance” in their lives. This perspective was consistently shared by both male and female therapists. For instance, one therapist noted, “Even when there is no time in my day for exercise, I will take a long walk”. This approach was seen as a way to ensure physical movement and engage in an activity dedicated to themselves. One participant emphasized the importance of managing burnout for the sake of client safety and support, stating, “You cannot pour from an empty cup”. In addition to embracing a healthier and more energetic lifestyle, some participants highlighted the use of relaxation techniques such as deep breathing exercises and progressive muscle relaxation. Implementing these techniques before and after sessions was thought to help them connect with their emotions, regulate their feelings, and manage their thoughts:

“Engaging in deep breathing exercises helps me become aware of my symptoms. When I notice them, I take a moment to breathe and assess myself. I ask, ‘What am I feeling? Where do I feel it in my body?’ This process helps me connect with my emotions, and I then utilize techniques such as progressive muscle relaxation or safe place meditation”.

## 4. Discussion

### 4.1. Discussion of Main Findings

The aim of this study was to provide insights into the experience of burnout among integrative psychotherapists and to explore the strategies participants employ to cope with this experience. Consistent with previous research [9], our participants confirmed that the pandemic increased their workload, prompting many individuals to seek professional support. However, some participants emphasized that this increased workload intensified the pressure they felt, leading to negative repercussions on their work productivity and making them more vulnerable to burnout. The novel context of the COVID-19 pandemic amplified the challenges faced by practitioners [8,10], making the already demanding role of the therapist even more taxing. For some in our study, the unprecedented nature of the crisis and the heightened emotional needs of clients appeared to lead to deeper emotional involvement in their work, which, while rewarding, also left them more susceptible to burnout. The unique pressures of this period thus represent a significant finding, highlighting how extraordinary circumstances can exacerbate existing stressors and introduce new ones within the therapeutic profession.

Some participants experienced deeper emotional involvement in their work, not only as an increased desire to help clients but also as a measure of their self-worth. Specifically, participants expressed an intense sense of responsibility toward their clients and a perceived obligation to assist them, which heightened the pressure to provide help and led to overinvolvement in therapeutic relationships. This overinvolvement and eagerness to support clients were closely tied to the therapists’ sense of self-worth. Notably, novice therapists indicated that observing progress in their clients validated their capabilities and worthiness as professionals, which was a persistent concern that exacerbated their burnout. Feelings of inadequacy and a strong desire to prove their worth are common among psychotherapists [17,18]. Particularly among novice therapists, who are striving in their early years to develop their competence and establish themselves in their profession [7], this strong desire to help clients and demonstrate their worth may increase their vulnerability to experiencing burnout. As such, the pandemic, along with the increased workload and the therapists’ need to accommodate clients’ needs, further heightened this pressure. Their belief that successfully helping clients would validate their professional competence was identified in this study as a key factor contributing to their increased vulnerability to burnout.

Our study corroborates that fatigue, overwhelm, and disconnection from clients are key symptoms of burnout, aligning with the existing literature [3,5,20,21]. Our findings, however, lend empirical support to the reconceptualization of burnout syndrome as encompassing four key symptom categories: exhaustion, mental detachment, and impairments in both cognitive and emotional functioning [6]. Specifically, participants described exhaustion as a persistent state characterized by chronic fatigue, headaches, and sleep disturbances. Mental detachment manifested through a disengagement from work and the development of negative attitudes toward clients. Cognitive impairments were evident, with some therapists reporting difficulties in creativity, decision-making, and session planning. Emotional impairments were reflected in a diminished capacity for empathy and emotional support, which in turn compromised the therapeutic alliance and client progress. Additionally, a novel symptom related to emotional impairment that emerged from our study was the fact that several participants reported experiencing a sense of numbness or a “freezing” sensation during sessions. This was perceived as a form of paralysis, significantly hindering their ability to continue sessions effectively. Therefore, burnout should be understood as a multifaceted syndrome that goes beyond the traditional understanding of emotional exhaustion. The inclusion of mental detachment, cognitive impairments, and emotional dysfunctions provides a more complete picture of how burnout manifests in professionals, highlighting novel symptoms that were not previously emphasized. This broader symptomatology may better capture the range of challenges faced by those experiencing burnout, particularly in demanding fields like psychotherapy.

In terms of coping strategies, personal therapy, and clinical supervision were proposed in our study as key supportive elements in managing burnout among integrative psychotherapists. Some participants suggested that increasing the frequency of both personal therapy and clinical supervision during periods of burnout while simultaneously decreasing client workloads could serve as a counterbalance to the pressures involved. These two supportive mechanisms are posited as mediating factors that buffer the effects of burnout and contribute to the overall well-being of therapists [15,16]. They not only assist therapists in managing their symptoms and caseloads, thus mitigating the negative impact of therapist burnout on clients, but, as our study highlights, they can also help therapists understand their own humanity. While burnout is common among psychotherapists [1], those experiencing it may struggle to recognize its prevalence or may feel that they should not encounter such challenges. However, personal therapy and clinical supervision may be able to facilitate the realization of the commonality of burnout or negative feelings that therapists might harbor during this period. 

In addition to these supportive mechanisms, participants noted that communicating with fellow therapists who have undergone similar experiences provides significant assistance. However, participants emphasized the critical need for support from their professional bodies. Specifically, they advocated for an organized effort by these bodies to bring professionals together and establish support groups that would facilitate knowledge sharing and prepare them to handle unexpected incidents. The need for support from professional bodies was perceived as becoming particularly evident during the pandemic, when therapists lacked the necessary support and knowledge to manage their work effectively. The pandemic and subsequent lockdowns compelled therapists to alter their professional routines and adapt to new realities, such as shifting to online sessions, while balancing family challenges and changes [10]. This period appears to have highlighted the insufficient support that psychotherapists may experience and underscored their need for a professional body that provides guidance and knowledge during unforeseen circumstances such as the pandemic. The current study advocates for systemic changes within professional bodies and healthcare organizations. Participants’ calls for structured support groups and enhanced knowledge-sharing initiatives during crises like the pandemic underscore the role of organizational leadership in fostering preparedness among psychotherapists. Promoting a culture of well-being within these organizations involves implementing practices that prioritize mental health, work–life balance, and continuous professional development. This includes creating supportive work environments where psychotherapists feel valued, have access to mental health resources, and are encouraged to engage in self-care practices without stigma. Additionally, it involves regular opportunities for peer support, supervision, and open dialogue about the challenges of the profession. By embedding these practices into the organizational culture, healthcare institutions can safeguard against burnout and promote sustainable professional practice, ensuring that therapists are equipped to provide the highest quality of care.

Participants also mentioned personal development, inner dialogue, relaxation techniques, and attentiveness to their body cues as strategies to manage burnout. Research has identified bodily aches, weight loss, and an inability to focus as some of the primary symptoms through which burnout manifests [22,23]. Therefore, therapists should remain vigilant to the signals their bodies are sending and seek to alleviate these symptoms by attending to their self-care and physical well-being during times of need. Furthermore, adopting a healthier lifestyle, including nutritious food, regular exercise, and a specific routine that prioritizes the therapists’ needs, was proposed as essential in managing burnout. The role of lifestyle factors in the development and management of burnout symptoms cannot be overstated. It has been reported that individuals suffering from burnout may be prone to emotional eating [24], and burnout may be associated with the frequent use of unhealthy food substances [25]. Conversely, healthy eating has been found to protect against burnout, as frequent consumption of healthy food items is associated with lower levels of burnout symptoms [26]. As such, our findings emphasize the importance of a balanced, healthy diet and routine in promoting work well-being and mitigating burnout. Finally, one participant notably highlighted her inclination to explore botany and herbal remedies as natural approaches to managing burnout. This finding suggests that some psychotherapists may seek alternative, holistic strategies to cope with burnout, reflecting a broader interest in integrating natural and complementary therapies into their self-care practices. It also underscores the diversity of coping mechanisms among therapists and indicates that personal preferences and beliefs may significantly influence their chosen methods of managing professional stress.

### 4.2. Methodological Considerations

This study involved a diverse group of participants with varying years of experience and from different age groups, which enriched the exploration of burnout and resilience and contributed to the overall depth of the findings. However, several limitations need to be acknowledged, particularly regarding the potential lack of generalizability and other methodological constraints. Firstly, the predominance of Greek participants in the sample may limit the applicability of the findings to other cultural contexts. Cultural factors may significantly influence both the experience of burnout and the strategies employed to cope with it. Therefore, the results may not fully reflect the experiences of therapists practicing in different cultural environments where distinct pressures, norms, and support systems might exist.

Additionally, the sample encompassed 14 women and only three men. This gender imbalance may affect the generalizability of the findings, particularly in relation to gender-specific experiences of burnout and coping strategies. Given that burnout can manifest differently across genders, with men and women potentially adopting different strategies for managing stress, the findings may not fully capture the diversity of experiences present in a more gender-balanced sample. Future research should aim to include a more balanced representation of male and female therapists to better understand how gender dynamics influence burnout and resilience. The study also focused exclusively on integrative psychotherapists. While this provided valuable insights into the burnout experiences and coping strategies within this specific therapeutic orientation, the findings may not be representative of therapists from other approaches. Different therapeutic models might emphasize varying aspects of self-care, supervision, and client interaction, potentially leading to diverse burnout experiences and strategies for managing this experience. This limitation suggests that the study’s findings should be interpreted with caution when considering their applicability to therapists from other orientations.

An additional limitation of this study is that the participating psychotherapists reported having experienced burnout in the past. As the study relied on participants’ recollections of their past experiences, there is a possibility of recall bias, which may have affected the accuracy or completeness of their accounts. Consequently, the findings are based on self-reported experiences of burnout rather than on data obtained through objective measurement tools.

Moreover, the qualitative nature of this research, while providing rich, in-depth data, inherently limits the ability to generalize the findings to a broader population. Qualitative research prioritizes depth over breadth, and as such, the experiences and strategies discussed here may not be representative of all integrative psychotherapists, let alone therapists in general. Given these considerations, future research should aim to include a more diverse sample of therapists, encompassing various cultural backgrounds, therapeutic orientations, and a more balanced gender distribution, to achieve a more comprehensive understanding of burnout and resilience. Additionally, employing a mixed-methods approach could enhance the study’s generalizability by complementing qualitative insights with quantitative data, thereby providing a more robust analysis of the factors influencing therapist burnout across different contexts.

## 5. Conclusions

This study has shed light on the pervasive issue of burnout among integrative psychotherapists, highlighting its complex manifestations and the coping strategies employed by practitioners. The findings underscored the significant impact of personal development, self-awareness, and lifestyle factors in managing burnout symptoms. Moreover, the study revealed the critical role of professional support mechanisms, such as clinical supervision and organizational interventions, in mitigating burnout and promoting therapist well-being.

The implications of these findings extend beyond the field of psychotherapy. Professionals in other healthcare disciplines, such as nurses and healthcare providers, who similarly navigate high-stress environments and emotional demands, may benefit from adopting similar strategies. By prioritizing self-care, cultivating self-awareness, and implementing supportive organizational practices, professionals can better manage the challenges associated with their roles. Moving forward, future research should explore burnout experiences across diverse therapeutic modalities and cultural contexts to broaden our understanding of effective interventions. This includes investigating the impact of burnout on patient care outcomes and organizational effectiveness, advocating for policies that prioritize healthcare workers’ mental health, and fostering a culture of support and resilience across healthcare settings. By prioritizing therapist mental health, healthcare systems can cultivate environments conducive to high-quality care and sustainable professional practice, ultimately benefiting both practitioners and the clients they serve.

## Figures and Tables

**Table 1:** Participants’ demographics.

Participants	Nationality	Gender	Age	Years of Practice
P1	Greek	Female	56	25
P2	Greek	Female	29	3
P3	Greek	Female	50	20
P4	Greek	Female	29	4
P5	Greek	Female	48	25
P6	Serbian	Female	28	3
P7	Greek	Male	32	9
P8	Greek	Female	34	4
P9	Greek	Female	32	3
P10	Greek	Female	37	9
P11	Greek	Male	33	11
P12	Greek	Female	28	4
P13	Greek	Female	52	4
P14	Dutch	Male	27	1
P15	Greek	Female	33	9
P16	Greek	Female	29	3
P17	Greek	Female	36	2

## Data Availability

Data can be made available upon reasonable request.
